# Assessing Quadriceps Muscle Contraction Using a Novel Surface Mechanomyography Sensor during Two Neuromuscular Control Screening Tasks

**DOI:** 10.3390/s23136031

**Published:** 2023-06-29

**Authors:** Shannon E. Linderman, Donna Moxley Scarborough, Ryan Aspenleiter, Hannah S. Stein, Eric M. Berkson

**Affiliations:** 1 Department of Orthopaedic Surgery, Massachusetts General Hospital, Boston, MA 02114, USA; shannon-linderman@uiowa.edu (S.E.L.); steinh@upstate.edu (H.S.S.); eberkson@mgh.harvard.edu (E.M.B.); 2 FIGUR8, Inc., Boston, MA 02116, USA; ryan.aspenleiter@figur8tech.com

**Keywords:** surface mechanomyography, surface electromyography, muscle excursion

## Abstract

Electromyography (EMG) is the clinical standard for capturing muscle activation data to gain insight into neuromuscular control, yet challenges surrounding data analysis limit its use during dynamic tasks. Surface mechanomyography (sMMG) sensors are novel wearable devices that measure the physical output of muscle excursion during contraction, which may offer potential easy application to assess neuromuscular control. This study aimed to investigate sMMG detection of the timing patterns of muscle contraction compared to EMG. Fifteen healthy participants (mean age = 31.7 ± 9.1 y; eight males and seven females) were donned with EMG and sMMG sensors on their right quadriceps for simultaneous data capture during bilateral deep squats, and a subset performed three sets of repeated unilateral partial squats. No significant difference in the total duration of contraction was detected by EMG and sMMG during bilateral (*p* = 0.822) and partial (*p* = 0.246) squats. sMMG and EMG timing did not differ significantly for eccentric (*p* = 0.414) and concentric (*p* = 0.462) phases of muscle contraction during bilateral squats. The sMMG magnitude of quadriceps excursion demonstrated excellent intra-session retest reliability for bilateral (ICC_3,1_ = 0.962 mm) and partial (ICC_3,1_ = 0.936 mm, *n* = 10) squats. The sMMG sensors accurately and consistently provided key quadriceps muscle performance metrics during two physical activities commonly used to assess neuromuscular control for injury prevention, rehabilitation, and exercise training.

## 1. Introduction

The ability to assess qualities of a muscle as it contracts may be advantageous for clinicians’ management of athletes’ performance and injury rehabilitation and readiness to return to sport after injury [1]. Quantifying the timing of muscle activation and the magnitude of a muscle contraction may provide insight into the coordination of muscle groups during dynamic movement and may help to direct treatment for improving performance and injury recovery/avoidance [2]. Sensor technology offers various methods of capturing a range of metrics during muscle contraction. Dynamic surface electromyography (sEMG) is the clinical standard for assessing the electrical signal identifying the timing of muscle activation [3]. However, there are several data collection and analysis challenges that limit the utility of EMG for wide-spread neuromuscular control assessments during dynamic movements [4]. Therefore, the application of novel sensors that can provide clinically useful information about muscle contraction in an easy and accurate manner will be advantageous for neuromuscular evaluation and rehabilitation.

The myoelectrical signal detected by sEMG is subject to impedance from sweat and hair and requires specific placement over the muscle of interest, which can complicate athlete assessments [3]. Signal processing is time-consuming and labor-intensive to interpret, and there is little standardization of the analysis methodology [4]. There are concerns regarding the reliability and validity of sEMG signal magnitudes and processing artifacts, each of which require interpretation by an experienced reviewer [5]. sEMG is also subject to signal crosstalk or interference from other neighboring muscles [6]. In response, investigators have attempted to evaluate additional measurement devices that can be used in conjunction with sEMG to assess muscle function and overcome some of these common critiques. One such method is to compare the sEMG signal magnitude during a functional activity to that of a maximal voluntary isometric contraction (MVIC) [7]. While this is a well-known normalization method, true subject effort has been shown to impact the validity of the results [8,9].

Mechanomyography (MMG), a methodology for assessing the mechanical activity of muscle during contraction, has recently gained attention as a counterpart or alternative to EMG [10]. MMG sensors include technologies such as a mechanical transducer [11], accelerometer [12], electrostatic microphone [13], or laser [14] to capture the physical excursion of muscle fibers during a contraction. These systems may provide information on the physical properties of a muscle contraction, but many are limited in application during dynamic activities. Therefore, there is a knowledge gap between the properties of muscle contraction when in a static posture compared to during dynamic movement.

The original technologies categorized as MMG are non-invasive tools that record muscle activity via the detection of low-frequency lateral oscillations in skeletal muscle fibers caused by voluntary or stimulated isometric or dynamic muscle contraction [10]. These oscillations can be captured by transducers such as accelerometers [12], piezoelectric contact sensors [4], electrostatic microphone [13], and laser excursion sensors [14]. Accelerometers capture the acceleration of skin/muscle excursion caused by pressure waves which surge through the surface of the skin following muscle contraction [10]. Piezoelectric contact sensors capture muscle contraction when an applied force, pressure, or strain from a contraction leads to a directly proportional charge excursion on the piezoelectric element of the sensor [4,15]. Electrostatic microphones capture changes in the frequency of pressure or sound waves that occur because of oscillating muscle fibers during muscle activation [16]. Laser excursion sensors measure the low-frequency oscillation of a superficial muscle belly [14]. Tensiomyography (TMG) captures the radial excursion of a muscle using a retractable transducer placed perpendicular to the muscle belly during a maximal involuntary muscle contraction elicited by electrical stimulation [10].

**Figure 1:**
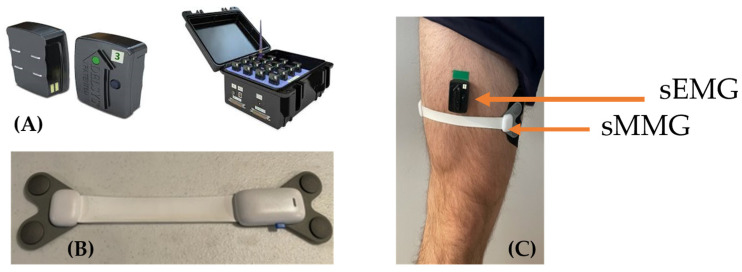
sEMG (**A**) and sMMG (**B**) sensor placement across the quadriceps muscle (**C**).

This study implements a new MMG sensor known as Surface MMG (sMMG) that, when placed over a muscle belly, outputs the linear excursion of the muscle during its contraction throughout dynamic activity (Figure 1). sMMG sensors are novel wearable devices comprised of a flexible electroactive polymer sensor that is applied to the skin over the muscle group of interest. These sensors expand (or stretch) when applied perpendicular to the muscle, activating and recognizing the physical muscle contraction through the change in the length of the sensor. The user is provided with information on the timing of when the physical contraction begins and ends, as well as the magnitude of the physical change in the muscle beneath it.

**Figure 2:**
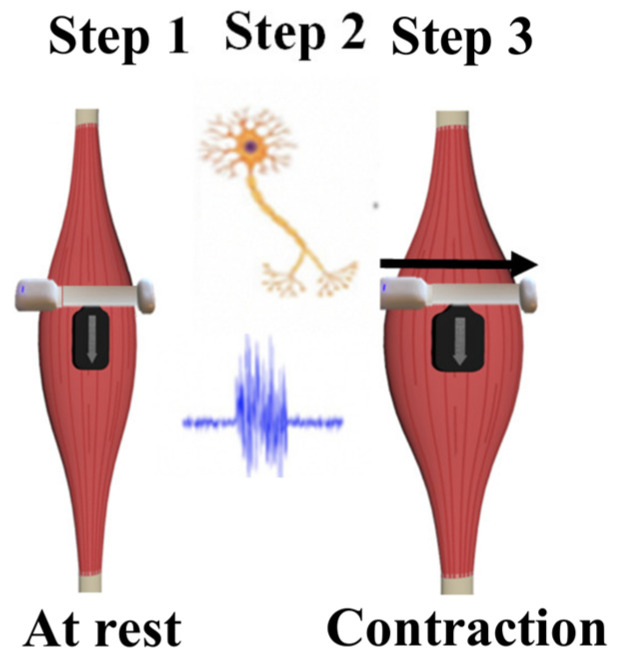
Steps from (1) cognitive decision making to move the limb, followed by (2) an electrical impulse transmitted down an axon and resulting myoelectric activity, leading to (3) physical contraction of the muscle and eventual movement.

Since MMG analyzes physical muscle deformation as opposed to the myoelectrical signals evaluated by EMG, it has demonstrated complementary use alongside sEMG for dynamic applications (Figure 2). Initial prototype testing of the sMMG sensor reported a strong correlation of contraction duration time with surface EMG and force production measured from a dynamometer [17,18]. However, it is unknown if novel sMMG sensors are reliable when applied to the dynamic activities desired by clinicians for assessing the timing of dynamic contractions or the reliability to quantify changes in the muscle output associated with contraction.

The purpose of this study was to investigate the timing patterns of muscle contraction and magnitude using sMMG sensors and compare time events to those collected through traditional clinical EMG during the bilateral squat and repeated unilateral squat. Our hypotheses are as follows: (1) There will be no significant difference in the timing duration of quadriceps muscle contraction between EMG and sMMG during both activities. (2) There will be no significant difference in the timing duration of concentric and eccentric quadriceps muscle contraction between EMG and sMMG for bilateral deep squat. (3) The magnitude of sMMG excursion will demonstrate good-to-excellent repeatability between trials of the bilateral deep squat and repeated unilateral partial squat activities and will offer repeatability similar to or greater than sEMG %MVIC measures of peak contraction.

## 2. Materials and Methods

### 2.1. Participants

Fifteen healthy, active individuals (mean age = 31.7 ± 9.1 y, height: 1.75 ± 0.10 m, weight: 75.43 ± 17.14 kg) participated in this cross-sectional study. Eight males and seven females provided informed consent to participate in this institutional-review-board-approved study. All participants had no history of lower extremity and/or back surgery or fracture or neurological disorder that influences movement patterns. Each person was required to be free of self-reported injury three months prior to enrollment.

### 2.2. Equipment

The sMMG sensor data were collected using a mobile app on an iOS device and transmitted at 50 Hz via Bluetooth low energy (FIGUR8, Inc., Boston, MA, USA). sMMG sensors detect the physical output from a muscle as it changes shape during contraction. Using a flexible component placed across the surface of a muscle bulk, the sensors record a change in capacitance that corresponds to an output in mm (100 mm range, resolution 100 µm) (Figure 1). This output represents the muscle bulk excursion as that muscle is activated during contraction and movement.

sEMG sensor data were collected at 120 Hz and streamed via a Bluetooth connection to a laptop (Delsys Trigno Avanti sensor, Natick, MA, USA). Electromyography detects the minute electrical signal that is produced by contracting muscles. EMG sensors are placed directly on top of clean and dry skin superficial to the muscle bulk of interest and record the spatial summation of action potentials produced to the active motor units and associated muscle fibers within a contracting muscle (Figure 1) [19].

The sMMG and sEMG sensors were simultaneously applied to the largest portion of the quadriceps muscle bulk of the right leg of each participant using adhesive tape. The skin was prepped as per standard protocol for electromyography with an alcohol wipe to clean the skin surface.

### 2.3. Assessment Activities

The participants were verbally cued to begin each assessment activity. Prior to the start of movement for each activity, subjects were instructed to gently tap their thigh in order to create a characteristic signal to assist with time stamp alignment between the sEMG and sMMG measurement systems and then pause for several seconds before initiation movement.

#### 2.3.1. Calibration

The participants first performed a standing calibration trial to record the baseline physical and electrical output of their quadriceps at rest. The subjects stood at rest with the sensors in place with their feet placed shoulder width apart for five seconds.

#### 2.3.2. Maximum Voluntary Isometric Contraction (MVIC)

After a brief warmup, the participants were instructed to perform an activity to illicit a maximum voluntary isometric contraction (MVIC) of the quadriceps [9]. Participants began seated with 1/3 of their hamstrings’ muscle bulk supported in a chair with their leg extended out in front of them with 90° hip flexion and their contralateral leg placed flat on the floor. With one hand, the tester completely supported the subject’s outstretched leg directly under the distal thigh so that their quadriceps were relaxed, leaving their other hand free to hold the dynamometer. The participants were verbally cued to begin and were instructed to push as hard as possible for three seconds against the hand-held dynamometer that was placed the width of three fingers above the ankle joint line [20,21,22]. The recorded peak electrical activity of the quadriceps recorded during the MVIC task was then determined for each subject and was used as a reference value to normalize the EMG magnitude for each subject in the study.

#### 2.3.3. Bilateral Deep Squat

**Figure 3:**
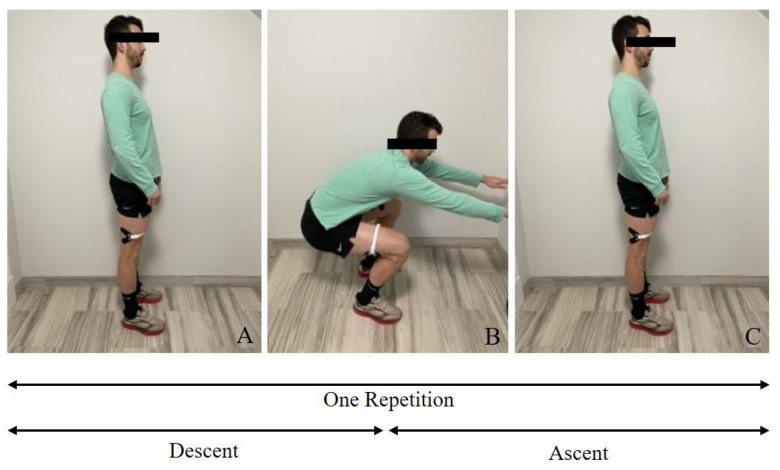
Sagittal view of starting position, descent (**A**,**B**) and ascent (**B**,**C**) to return to starting position during the bilateral deep squat task.

All the participants were instructed to begin standing with their feet placed shoulder width apart and arms extended straight out in front of them at shoulder height (Figure 3). The participants were then asked to perform an unassisted bilateral squat at a comfortable and self-selected pace and to squat to the furthest possible depth without allowing their hamstrings and gastrocnemii to make physical contact while their heels remained firmly planted on the ground. The participants were also instructed to briefly pause at the nadir of the squatting motion before returning to the initial standing position. The participants performed one squat during each trial and completed a total of three trials.

#### 2.3.4. Repeated Unilateral Partial Squat

**Figure 4:**
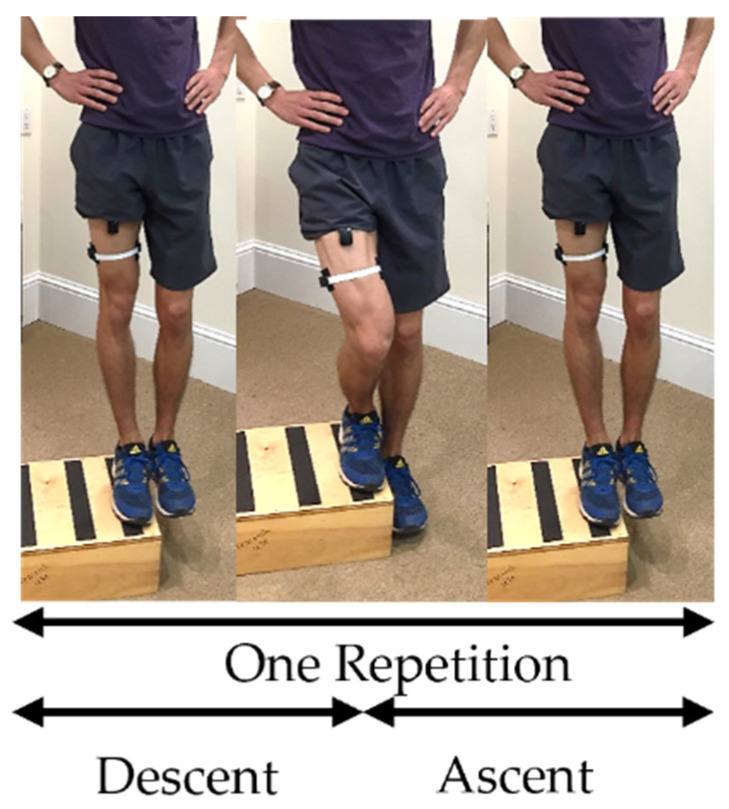
One repetition of a repeated unilateral partial squat (RUPS).

A subset of ten participants then also performed three trials of a repeated unilateral partial squat activity. For this task, the subjects were instructed to begin standing on one leg on the edge of a 20 cm box with hands placed on their hips and the contralateral limb hanging freely off the side of the box. The participants then squatted on the supporting limb and lowered the contralateral limb as close to the floor without ground contact before returning to the starting position. Five repetitions of the partial squat activity were repeated per trial (Figure 4). Each participant performed one practice trial. Any trials where the participants lost their balance and touched the side of the box or the ground were excluded. Each participant attempted to perform three successful trials on their dominant supporting leg. Participants unable to complete three successful trials of the activity were excluded from the analyses. Analyses were planned for only the middle three of the five partial squats per trial when the participants are the most balanced during the activity.

### 2.4. Data Processing

**Figure 5:**
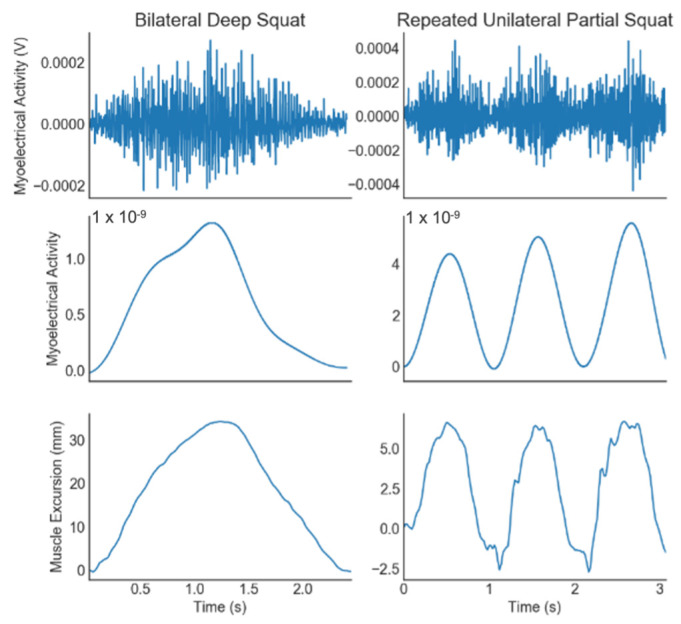
Example of data analysis steps for comparison between electromyography (EMG) and surface mechanomyography (sMMG).

Raw sMMG data were used in all the analyses while signal processing was applied to EMG data (Figure 5). A rectified Teager–Keiser energy operator (TKEO) was first applied to the EMG data [23]. EMG data were then processed with a low-pass 6th order Butterworth filter prior to the timing analyses. To account for the asynchronous recording of the two systems, trials were cropped based on the activation and deactivation of the quadriceps. Activation was determined as three times the standard deviation of the calibration activity, while deactivation was defined as a return to baseline activity level. Contraction duration was calculated by subtracting the activation time from the deactivation time. Further, eccentric quadricep contraction for the bilateral deep squat and the unilateral partial squats was measured by subtracting the activation time from the peak time, while concentric contraction was the deactivation time minus the peak time. Eccentric and concentric contraction timing was based on sEMG parameters, as opposed to visual observation, since changes in muscle activation that will be detected by EMG are required to be performed first in order to dictate the visible changes in the direction of the subject’s movement [24].

### 2.5. Data Analysis

The time point of muscle activation and deactivation was set at a threshold value three times the standard deviation of the standing calibration trial above the minimum value for the dynamic trials. The timepoints of muscle activation, peak contraction, and deactivation were calculated using both sEMG and sMMG.

The magnitude of peak quadriceps muscle bulk excursion as detected by sMMG for each trial was also recorded along with the magnitude of the peak value of the processed sEMG signal for each subject. sEMG magnitude was reported as a percentage of the recorded MVIC trial for each subject.

Statistical analyses for comparison of time signatures between modalities include paired T-tests. Within-session reliability was assessed via a two-way mixed effects intra-class correlation reliability model. The threshold of statistical significance was established at *p* < 0.05.

## 3. Results

### 3.1. Timing of Muscle Contraction

There was no significant difference in the timing of the total duration of quadriceps contraction between sEMG (mean = 2.552 ± 0.589 s) and sMMG (mean = 2.560 ± 0.576 s) during the bilateral deep squat, *p* = 0.822. The duration of quadriceps contraction during the descent phase of the deep squat (eccentric contraction, *p* = 0.414) and the ascent phase (concentric contraction, *p* = 0.462) did not differ significantly between modalities (Table 1). The timing of average quadriceps contraction during a repeated unilateral partial squat also did not differ significantly between sEMG (mean = 1.747 ± 0.569 s) and sMMG (mean = 1.763 ± 0.593 s), *p* = 0.246 (Table 2).

### 3.2. Detection of Peak Quadriceps Contraction

**Figure 6:**
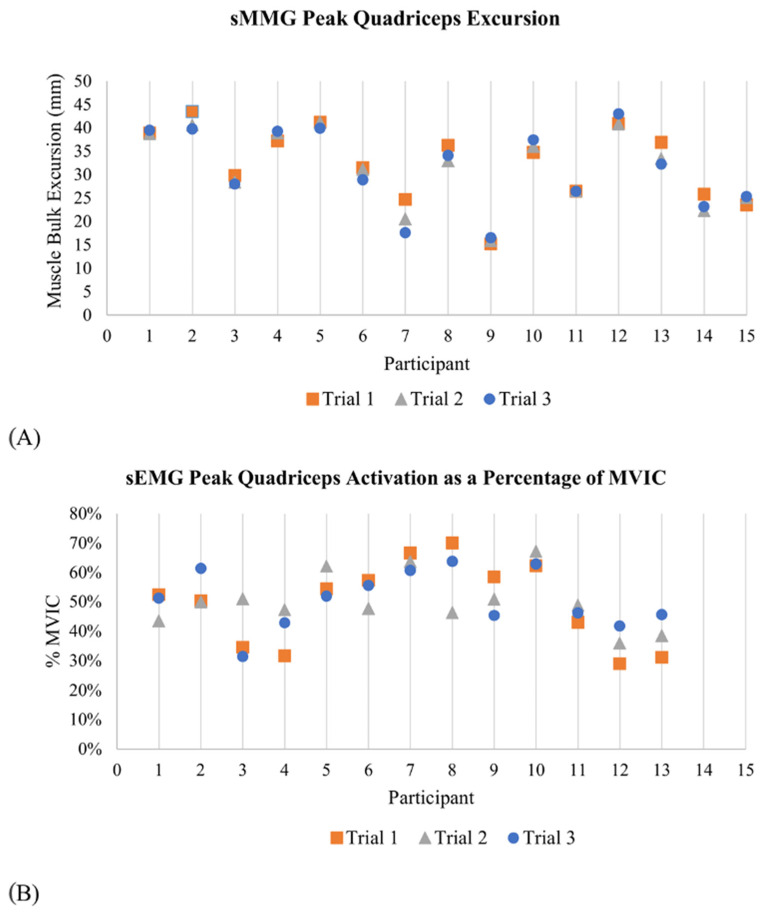
Intrasession reliability of peak quadriceps physical activation as measured by sMMG (**A**) and electrical activation as measured by EMG (**B**) during a bilateral squat task.

The magnitude of peak physical muscle bulk excursion during the bilateral squat detected by sMMG demonstrated excellent within-session retest reliability across all three trials for each participant (ICC_3,1_ = 0.962 mm), Figure 6, based on the reliability thresholds established by Koo et al. [25]. The mean quadriceps excursion was 31.824 ± 8.027 mm with an average within-subject variance of 2.530 mm. Average peak quadriceps electrical activity detected by sEMG during the bilateral squat task was 51.78 ± 11.92% of a maximum voluntary isometric contraction (MVIC) with an average within-subject variance of 0.68%. Percentage of MVIC values demonstrated moderate within-session retest reliability across all three trials for each participant (ICC_3,1_ = 0.528), Figure 6 [25].

**Figure 7:**
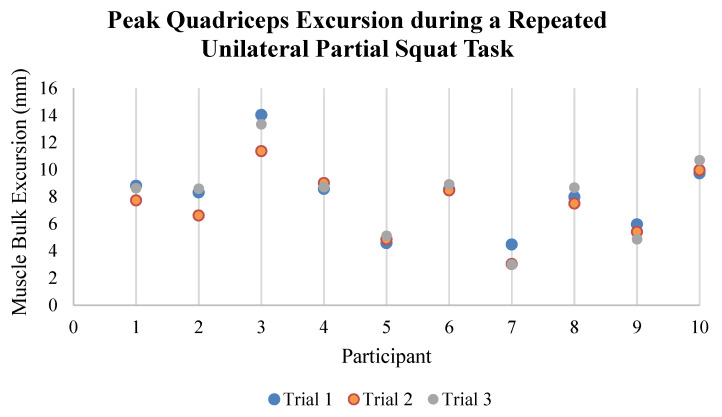
Intrasession reliability of peak quadriceps excursion during the third repeated unilateral squat repetition across the three sampled trials for each participant.

Excellent intra-session retest reliability for the magnitude of quadriceps excursion was also observed for the repeated unilateral partial squat activity (ICC_3,1_ = 0.936 mm, *n* = 10); Figure 7. The mean peak quadriceps muscle bulk excursion across all RUPS trials was 7.948 ± 2.719 mm. The within-subject variability in the magnitude of quadriceps excursion during the RUPS task was 0.600 mm. Average peak quadriceps electrical activity detected by sEMG during the RUPS task was 44.24 ± 18.89% of each subject’s maximum voluntary isometric contraction (MVIC) with an average within-subject variance of 0.57%. A percentage of MVIC values demonstrated good within-session retest reliability across three bilateral squats for each participant (ICC_3,1_ = 0.850, *n* = 10), Figure 6 [25].

## 4. Discussion

### 4.1. Overview of Findings

The study findings reveal similarities in time signatures between the sMMG and sEMG sensors for assessing key clinically relevant time points of quadriceps activation and deactivation during common neuromuscular assessment activities involving distinct eccentric and concentric phases of movement. The observation that the average within-subject variation in the timing of muscle contraction duration was not significantly different between the two modalities further increases confidence in these results. We also assessed the test–retest reliability of the sMMG sensor output metric of muscle bulk excursion. Based on the definitions by Koo et al., we were able to establish excellent reliability of this sMMG measurement type with ICC values ranging from 0.962 to 0.936 during two screening activities commonly used in athletic and clinical populations [25]. Furthermore, the intrasession reliability of the sMMG physical measurement of the magnitude of peak muscle bulk excursion displayed greater reliability than the magnitude of the EMG electrical signal measure reported as %MVIC while using simultaneous sensor data collection across two activities.

### 4.2. Timing Literature (Discussion of Contraction Duration Timing Results—Hypothesis 1)

The potential ability to reliably detect the duration of muscle contraction from a mechanical signal has been suggested using MMG methods throughout the literature [10]. Duration of contraction as measured during isometric contractions via tensiomyography (TMG) has been identified as having moderate-to-high ICC values (0.70–0.98) [26]. The validity of such measures has also been established via comparison to the clinical gold-standard of EMG, and timing of contraction is one of the most frequently extended measures to clinical and athletic environments.

The muscle contraction timing results of this study directly align with prior findings and expectations. During muscle activation, myoelectrical activity slightly precedes physical muscle deformation [3]. However, due to the extreme speed of neuromuscular signal transfer that creates a physical contraction, these differences in signal transmission are not expected to result in a significantly different overall duration of muscle contraction. Mechanical and electrical signal types are thus both required for gross muscle activation, leading to the physical contraction of the muscle bulk and movement.

Similarly, although different sensor types are used to measure the electrical and physical signal types that contribute to muscle activation, the overall duration of the contraction timing results obtained from both sensor types is not expected to differ significantly. sEMG measures an electrical signal, the temporal summation of action potentials that initiates a muscle contraction, whereas sMMG measures the mechanical signal resulting from physical muscle contraction that occurs rapidly after electrical activation [27]. Therefore, it is expected that there will be a very slight, but non-significant, difference in the timing of muscle activation measured via sEMG and sMMG. There was no significant difference in the timing duration of quadriceps muscle contraction detected by EMG and sMMG sensors in this investigation, which supports the first hypothesis of this study and is consistent with these physiologic expectations. Therefore, advantages may exist in terms of being able to accurately measure the duration of muscle contraction using electrical and mechanical signals through the use of both EMG and sMMG sensors depending on the specific physiology of interest. For example, the mechanical signal of sMMG accounts for the time required to “take up” the muscle tendon system slack prior to force development [28,29,30]. Similarly, during muscle relaxation, the electrical signal being assessed involves the cessation of the propagation of the action potentials and electrochemical signaling, influencing actin myosin crossbridge release, whereas the mechanical signal is primarily involved with the return of the muscle fibers to their initial length [3]. The detection of contraction delays, such as time from onset of activation until 10% of max excursion, may be important for the assessment of muscle activation lag following surgery and the evaluation of rehabilitation progress [31]. However, such measures are currently not being routinely assessed in clinic environments using EMG or MMG technology alone and could perhaps benefit from a combined analysis [29,31].

### 4.3. Discussion of Time Point Detection Results (Concentric and Eccentric—Hypothesis 2)

The sMMG sensors displayed similar detection to EMG for the timing of muscle contraction during different phases of dynamic movement that incorporate distinct eccentric (squat descent) and concentric (squat ascent) muscle activation, which successfully addressed the second hypothesis of this study. During the muscle activation to peak contraction time period of the squatting tasks performed in this study, the quadriceps muscle undergoes eccentric contraction, where the muscle contracts while elongating under load to help lower the body into a squatting position [32]. Conversely, during the peak activation to deactivation time points of the assessed squatting tasks, the quadriceps muscle contracts concentrically and shortens under load to allow the individual to return to a standing position [32]. The detection of deficiencies and delays during a specific type of muscular contraction is important to clinicians for assessing muscle imbalances that may predispose individuals to injury, as well as for the design of post-surgical rehabilitation protocols [33]. Although this study helps to establish that sMMG sensors can be used to assess the timing of muscle contraction during dynamic movements involving concentric and eccentric contractions as opposed to static isokinetic holds required for TMG study, further work is required to assess the ability of sMMG sensors to accurately distinguish between different types of muscle contractions.

### 4.4. Reliability Testing

Excellent levels of repeatability for the sMMG metric of muscle bulk excursion were observed between two different functional tasks, which supports the third hypothesis of this study. The magnitude of the sMMG signal in the form of the mm of muscle excursion specifically displayed repeatable analysis during two common neuromuscular control screening activities that have been shown in the scientific literature to require highly repeatable patterns of muscle activation for successful completion [34,35,36]. Analysis of the magnitude of the sMMG physical measure of muscle bulk excursion also displayed more consistent results across three trials of the assessed physical tasks compared to maximum sEMG signal %MVIC magnitude analysis. This is consistent with the expectations given the inconsistencies of %MVIC EMG measurement across the literature. Therefore, the sMMG metric of magnitude of muscle bulk excursion may help to provide new, more reliable information for measuring the magnitude of muscle bulk activation during dynamic movements.

### 4.5. Comparison to EMG

The study results demonstrate the ability of sMMG sensors to both complement EMG by providing comparable timing data while also potentially improving upon certain aspects of current EMG data collection and analysis by providing more reliable %MVIC measurements. The evaluation of muscle contraction timing was able to be performed on raw sMMG data without any of the complex signal processing required of EMG analysis, and still yielded comparable results to EMG. Historically, EMG and traditional MMG testing has required complex set-up and time-intensive signal processing (EMG linear envelope rectification and filtering) prior to analysis by an experienced technician, which has limited the widespread adoption of this technology in both clinical and athletics screening [37]. sEMG results are dependent on the stable contact of EMG sensors to dry, clean skin [6]. Further, the nature of the assessed myoelectric signal is subject to a high degree of variability and is very sensitive to noise from motion artifacts and signal interference from electromagnetic sources or simultaneous motor unit activation of surrounding muscle groups during whole-body and complex movements [6,15]. sMMG analysis involving the detection of physical muscle excursion may be able to avoid some of the well-established data collection challenges of sEMG, including the need for the minimization of potential sources of signal interference that are heightened during dynamic activities.

### 4.6. Comparison to TMG

Since skeletal muscle functions as an approximately constant volume system, changes in muscle bulk deformation during contraction have been used as indicators of muscle contractile performance and behavior during tensiomyography testing, a form of mechanomyography [10]. In particular, the magnitude of radial muscle excursion during an isometric twitch contraction has been demonstrated to be one of the most reliable and valid TMG measures, demonstrating good–excellent test–retest intrasession reliability [26]. A recent systematic review by Martin-Rodriguez of nine TMG studies identified a high ICC (0.91–0.99) for maximum muscle excursion across the literature [26]. The degree of intrasession reliability of the maximum quadriceps excursion observed in this study of sMMG sensors bears similarities to other commonly observed lower extremity measures during the bilateral squat screening activity [34,35,36,38]. However, formal comparisons of the intra- and inter-session reliability of the muscle excursion measured by sMMG and TMG still needs to be addressed.

### 4.7. Potential Uses in the Future

The ability of the sMMG sensors to successfully detect dynamic contractions, as opposed to the stimulated isometric contractions employed in many other forms of mechanomyography, offers some distinct advantages. The ability of sMMG sensors to also detect specific contraction types is also of great interest to coaches and trainers since athletes performing sport-specific maneuvers may place greater emphasis on eccentric or concentric contraction and therefore have a greater specific injury risk and fatigue concerns involving a particular contraction type [39]. There is interest in assessing neuromuscular control during a specific type of muscle contraction as part of screening activities. However, this differentiation is currently difficult and time-consuming to quantitatively assess using other commercially available methods. Based on the current findings demonstrating similar timing results to EMG during movements with distinct quadriceps eccentric (i.e., lowering into squat) and concentric (i.e., rising from squat) contraction phases, future work is indicated to further explore the potential of sMMG sensors to reliably detect and distinguish between different type of muscle contraction during complex, sporting movements and fatiguing maneuvers.

sMMG sensor measures may also have potential for application in future analysis of musculoskeletal fatigue as part of during athletic and neuromuscular control screening tasks. As maximal excursion and velocity of contraction to 10% and 90% of maximal excursion has been suggested as an indirect marker for peripheral/superficial muscular fatigue [28,40], sMMG measures of maximum muscle excursion could have potential for injury prevention/monitoring and rehabilitation settings. Deficiencies in maximum quadriceps muscle excursion have also been suggested to serve as a risk factor for anterior cruciate ligament (ACL) injury in athletes [41]. Maximum quadriceps muscle excursion has also been used as a metric to monitor lower extremity changes in muscle activation during post-ACL reconstruction rehabilitation [42], as well as increases in training load [43,44]. Further work is warranted to evaluate associations between sMMG measures during neuromuscular control screening to longitudinal injury and rehabilitation outcomes and sport-specific performance metrics.

### 4.8. Limitations

Some limitations include this observational study’s limited sample size of 15 participants and subjects’ performance of three trials of each movement task. However, this initial proof-of-concept study supports the hypotheses established in a sample size in line with and larger than other mechanomyography sensor design pilot studies with comparison to EMG [4]. The potential application of the sMMG sensor during a common athletic assessment consisting of the bilateral deep squat and repeated unilateral partial squats suggests this new technology is a promising method for assessing neuromuscular control. Further study is required to determine the contribution of the individual quadriceps muscle bulks, such as the rectus femoris alone to the sMMG measure of quadriceps muscle excursion, along with a further large-scale comparison of both sensor intrasession and intersession reliability compared to the reliability of other measures of radial muscle excursion in the literature, such as TMG. Overall, this foundational study establishes the potential for the sMMG analysis of the timing of muscle contraction alongside sEMG and the assessment of muscle bulk excursion. It highlights the added benefits of sMMG, including a lack of required complex signal processing.

## 5. Conclusions

The results of this study support the ability of the sMMG sensor to accurately detect quadriceps contraction due to the substantial timing similarities with simultaneous EMG capture. Additionally, the sMMG sensor demonstrates the strong repeatability of muscle output for both repeated activities. Together, these results suggest that the FIGUR8 sMMG sensors may be helpful for assessing quadriceps muscle performance and training as part of quick, in-the-clinic neuromuscular control screenings for injury prevention, rehabilitation, and exercise training.

## Figures and Tables

**Table 1:** Average timing of quadriceps contraction during phases of the bilateral deep squat.

Sensor	Descent Phase (s)	*p*	Ascent Phase (s)	*p*
EMG	1.250 ± 0.470	0.414	1.302 ± 0.631	0.462
sMMG	1.317 ± 0.429		1.243 ± 0.238	

**Table 2:** Average timing of quadriceps contraction during repeated unilateral partial squat.

Sensor	Partial Squat 2	*p*	Partial Squat 3	*p*	Partial Squat 4	*p*
EMG	1.748 ± 0.584	0.17	1.769 ± 0.622	0.369	1.759 ± 0.576	0.945
sMMG	1.784 ± 0.618		1.796 ± 0.639		1.761 ± 0.609	

## Data Availability

Data available on request due to privacy restrictions.
